# Deep Learning–Based Choroidal Boundary Detection in Geographic Atrophy Using Spectral-Domain Optical Coherence Tomography

**DOI:** 10.3390/diagnostics16050737

**Published:** 2026-03-02

**Authors:** Elham Rahmanipour, Nasiq Hasan, Adarsh Gadari, James Whitley, Soumya Sharma, Shreyaa Lall, Cristian de los Santos, Elham Sadeghi, Sandeep Chandra Bollepalli, Kiran Kumar Vupparaboina, Mario J. Savaria, Jay Chhablani

**Affiliations:** 1 Immunology Research Center, Mashhad University of Medical Sciences, Mashhad 91779 48564, Iran; elhamrahmanipour@gmail.com; 2 Department of Ophthalmology, University of Pittsburgh, Pittsburgh, PA 15261, USA; nah278@pitt.edu (N.H.); adg199@pitt.edu (A.G.); whitley.james@medstudent.pitt.edu (J.W.); shreyaa@pitt.edu (S.L.); sadeghie@pitt.edu (E.S.); sab517@pitt.edu (S.C.B.); kkv@pitt.edu (K.K.V.); 3 NetraMind Innovations Inc., Pittsburgh, PA 15238, USA; 4 College of Medicine, The University of Toledo, Toledo, OH 43606, USA; soumya.sharma@rockets.utoledo.edu; 5 Fundacion Retina, Buenos Aires B1669, Argentina; hes.oftalmalogia.cs@gmail.com (C.d.l.S.); mario.savaria@gmail.com (M.J.S.); 6 Buenos Aires Macula Clinical Research, Buenos Aires B1669, Argentina

**Keywords:** geographic atrophy, swept-source OCT, deep learning, choroidal segmentation, automated image analysis

## Abstract

**Background/Objectives**: To evaluate the challenges and limitations of a deep learning model for automated choroidal boundary detection in eyes with geographic atrophy (GA) using spectral-domain OCT (SD-OCT), and to assess the workflow efficiency of an AI-assisted manual verification approach. **Methods**: In this retrospective study, total 5723 scans (Heidelberg Spectralis) with GA were analyzed. A previously validated tool (NMI ChoroidAI) was used to segment the choroidal inner (CIB) and outer (COB) boundaries. We compared the “AI-assisted” workflow (automated segmentation followed by manual verification) against “manual segmentation only” in terms of accuracy and time consumption. Slice-wise boundary errors were graded as 0 (accurate), 1 (≤33% deviation), 2 (33–66% deviation), or 3 (>66% deviation). Outcomes included error rates and weighted F_1_ score (and precision where applicable). Total time for manual-only segmentation versus AI-assisted verification was recorded. -Interreader variability was assessed between the two readers using intraclass correlation coefficient. **Results**: For CIB, only 5.2% of B-scans showed any deviation (strictly accurate in 94.8%), with weighted F_1_ score 0.97 and precision 1.00. COB was more error-prone: 19.0% of B-scans showed deviation; however, when minor deviations were considered acceptable, COB acceptability increased to 94.2% (i.e., 5.8% remained >33% deviated). Only 13.2% of B-scans required minor manual correction. For a 97-scan volume, processing time decreased from an average of 7 h (manual only) to 45 min (AI + human verification), an approximate 90% reduction in manual effort. Inter-reader agreement was high (ICC 0.923 for CIB and 0.938 for COB). **Conclusions**: Although the deep learning model exhibits limitations in COB detection due to artifacts, it serves as a valuable assistive tool. Our model substantially reduces human effort, but mandatory human verification is required to correct boundary errors caused by hyper-transmission before use in clinical trials.

## 1. Introduction

The role of the choroid in age-related macular degeneration (AMD) pathophysiology remains debated, including the sequencing of choriocapillaris loss and retinal pigment epithelium (RPE) degeneration. In geographic atrophy (GA), the advanced stage of AMD, this temporal relationship between choriocapillaris dropout and RPE degeneration is correlated to longitudinal OCT-based quantification of choroidal structure [1]. Growing evidence highlights the role of the choroid in AMD and GA, reflecting its critical contribution to retinal metabolic support [2]. Choroidal thinning has been consistently reported in eyes with GA, while a distinct subset, referred to as pachychoroid GA, has also been identified [3]. Similarly, other choroidal biomarkers in geographic atrophy have also been explored, including the choroidal vascularity index (CVI) [4] and total choroidal volume, both of which have been shown to be reduced in GA [5].

Manual segmentation of the choroidal inner boundary (CIB) and outer boundary (COB) on high-density optical coherence tomography (OCT) volumes is prohibitively time-consuming for both clinical trials and routine practice. Precise delineation of these boundaries forms the foundation for deriving key choroidal biomarkers, including volumetric and sectoral choroidal thickness, choroidal volume, choroidal vascularity index, choroidal contour [6], and three-dimensional reconstruction of choroidal vascular architecture [7]. Deep-learning models can delineate the choroid reliably in many healthy eyes, but GA changes the imaging problem itself. With RPE loss, SD-OCT often shows marked hyper-transmission that can wash out the choroidal–scleral interface (CSI) or create “tailing,” so the boundary is no longer cleanly expressed in the intensity profile [8,9]. In this setting, accuracy reported in healthy or non-GA AMD cohorts may not carry over, because the disease introduces GA-specific contrast shifts and local ambiguities, most notably for COB detection [10,11]. Recent GA imaging work echoes this point, emphasizing that RPE loss fundamentally reshapes OCT contrast at the CSI in ways that can mislead automated boundary estimation [12].

In GA eyes, hyper-transmission can cause the CSI to become ambiguous and leading to COB errors such as boundary drift toward visually dominant “tailing,” discontinuities at hyper-transmission transitions, or local misplacement near vessel-related high-contrast streaks [8,9,12]. These errors are difficult to resolve autonomously because the CSI cue might be attenuated and intermittently absent under atrophic RPE which creates true signal ambiguity [12]. Therefore, compared with fully automated approaches, a human-in-the-loop workflow improves segmentation accuracy by applying targeted quality control to the highest-risk regions, COB segments within or adjacent to hyper-transmission zones and scans affected by motion or low signal, so that derived choroidal biomarkers remain clinically valid [8,9,10,11,12].

Currently, there is no automated algorithm available that can reliably segment the choroid in GA eyes without supervision. Given the persistence of these artifacts, the role of AI must be defined as assistive rather than autonomous. A “human-in-the-loop” approach is critical, where manual verification serves as a mandatory quality control step to correct errors caused by hyper-transmission, ensuring that efficiency does not come at the cost of clinical validity. To balance AI-related efficiency gains against the time burden of mandatory quality control, a triage-based review strategy such as rapid visual verification of most B-scans and manual correction of high-risk slices (e.g., hyper-transmission transition zones or scans with motion/low signal) should be applied. Relying on unsupervised segmentation in GA may introduce clinically relevant bias in derived choroidal biomarkers and can create spurious longitudinal change, potentially confounding imaging endpoints in longitudinal studies and interventional settings [8,9,10,11,12]. Therefore, at present AI should be used as an adjunct to human graders, reducing manual segmentation workload while preserving expert oversight, rather than as a fully autonomous solution. Fully unsupervised segmentation remains potentially risky, particularly in clinical trials where imaging endpoints may directly influence treatment paradigms. Accordingly, no AI-based method is expected to be 100% error-free in this setting. In this study, we evaluated a deep learning pipeline for the automated choroidal segmentation of GA eyes imaged with SPECTRALIS^®^ OCT system (Heidelberg Engineering, Heidelberg, Germany) [13,14]. Unlike lesion-detection work, we perform a “failure analysis” of boundary definition, hypothesizing that hyper-transmission artifacts affect COB more than CIB. We also quantify efficiency of an AI-assisted approach versus manual grading to see if time savings justify expert artifact correction.

## 2. Materials and Methods

### 2.1. Data and Imaging

This retrospective study included 5723 scans from 59 volumes (each 97 scans) with GA secondary to dry AMD. The study was conducted at the UPMC Vision Institute, from October 2023 to September 2024 following approval from the Institutional Review Board. The study adhered to the tenets of the Declaration of Helsinki. A “waiver of informed consent” was obtained considering the retrospective nature of the study.

### 2.2. Imaging Acquisition

Volumetric SD-OCT imaging was performed using the SPECTRALIS^®^ OCT system (Heidelberg Engineering, Heidelberg, Germany). Each volume covered a 6 × 6 mm area and consisted of 97 horizontal B-scans covering macular regions. This 6 × 6 mm cube format is consistent with prior Spectralis GA OCT datasets [13] and was used to provide standardized central macular coverage for GA assessment. The 97 B-scan density provides dense macular sampling for volumetric analysis and to reduce the likelihood of missing focal COB disruptions adjacent to hyper-transmission; this protocol also corresponds to the standard macular volume acquisition used at our center for GA assessments. It can scan up to 40,000 A-scans per second, has a wavelength of 870 nm and an axial resolution of about 3.9 µm in tissue. The SD-OCT software’s integrated scoring system, called the quality score or Q-score, evaluated the quality of the scans. Only scans with a quality score of 25 or higher on a scale of 0–40 met the criteria for inclusion in the analysis. This threshold was used to reduce low-signal scans that can destabilize boundary localization. For each volume, the SPECTRALIS^®^ system also acquired a co-registered infrared (IR) fundus image used for scan localization during acquisition. Scan quality was reviewed, and volumes with significant motion artifacts or signal loss were excluded from analysis. Motion artifacts were operationally defined as visible inter-scan misalignment or distortion (e.g., step-like discontinuities or duplicated/misaligned structures across adjacent B-scans) indicating fixation shifts during acquisition. Signal loss was operationally defined as regional or diffuse signal dropout and attenuation that obscured boundary visibility (particularly CSI/COB), making reliable delineation impractical.

**Figure 1:**
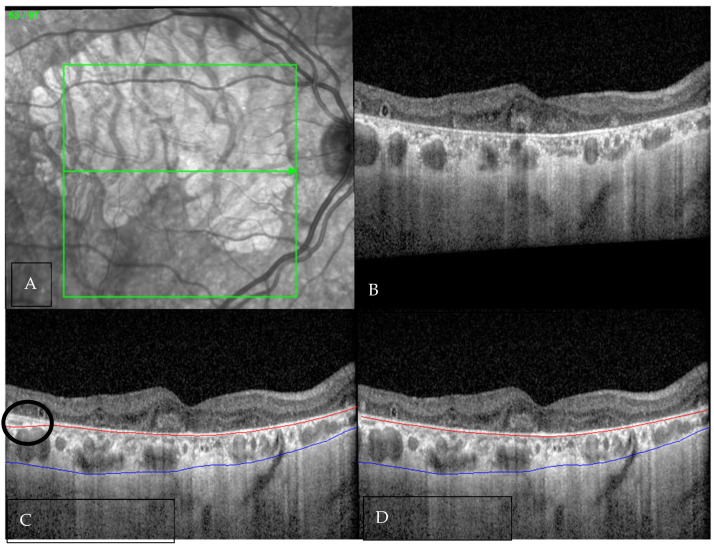
An 82-year-old male with fovea-involving geographic atrophy (GA). (**A**) Infrared reflectance image demonstrating the extent of GA and the location of the OCT scan. (**B**) Corresponding OCT B-scan showing diffuse hyper-transmission and the presence of outer retinal tubulations, consistent with GA. (**C**) OCT B-scan with automated segmentation of the choroid, demonstrating largely accurate choroidal boundary delineation with a minor inaccuracy (black circle) in the inner choroidal boundary (red line) at the temporal edge (black circle). The outer choroidal boundary (blue line) appears accurately segmented (**D**) Manually corrected segmentation with adjustment of the previously noted inaccuracy.

Two trained readers (SL and JW) independently assessed the OCT images and graded the degree of inaccuracy for each B-scan, while also recording the number of completely accurate B-scans within each volume. Inter-grader agreement for the assigned inaccuracy grades was quantified using the intraclass correlation coefficient (ICC; two-way mixed-effects model, absolute agreement). In cases of disagreement, adjudication was performed by a senior retina specialist (JC). A “completely accurate” B-scan was defined as Grade 0 (no deviation) for the evaluated boundary (CIB or COB) on that slice. Manual correction was performed in a subset of volumes, and the time required for full manual segmentation was compared with the time required for manual correction following automated segmentation (Figure 1). The subset was used only for workflow timing because full manual segmentation of all volumes was not feasible. Time comparisons were performed on three included volumes (Workflow Efficiency Assessment), and the accuracy/failure analyses were conducted on the full dataset.

### 2.3. Segmentation Model and Pipeline

We used the previously validated NMI ChoroidAI algorithm (NetraMind Innovations Inc., Pittsburgh, PA, USA), a deep convolutional neural network based on a ResUNet (Residual U-Net) architecture, to segment the choroidal inner and outer boundaries in the OCT volumes [15]. ResUNet was selected because its residual blocks and encoder–decoder skip connections are well suited to pixel-wise medical image segmentation and robust feature learning in noisy OCT data. The highest-probability pixel selection can be sensitive to local probability-map noise or discontinuities; therefore, we used minimal smoothing of the extracted boundary curve and mandatory overlay-based visual verification on the original B-scans before downstream analysis. The segmentation of choroid layer boundaries involves identifying two critical interfaces: the RPE–choroidal interface (CIB) and the choroid–sclera interface (COB/CSI). Manual delineation and correction of CIB and COB was performed using the manual segmentation feature on the NMI ChoroidAI tool.

Shadow compensation was applied to each B-scan to enhance visualization of the choroidal region. This preprocessing reduces attenuation/shadowing from overlying structures and improves deep-layer contrast, which can make the COB/CSI more visible; however, it cannot recover a CSI that is intrinsically indistinguishable due to GA-related signal washout. The preprocessed B-scan was then passed to the ResUNet, which produced pixel-wise probability maps for the CIB and COB. These probability maps were converted into continuous boundaries by selecting the highest-probability pixel for each boundary, followed by minimal post-processing (e.g., robust locally estimated scatterplot smoothing). For visualization and quality control, the predicted CIB and COB lines were overlaid on the original B-scans.

### 2.4. Performance Evaluation and Classification Metrics

**Figure 2:**
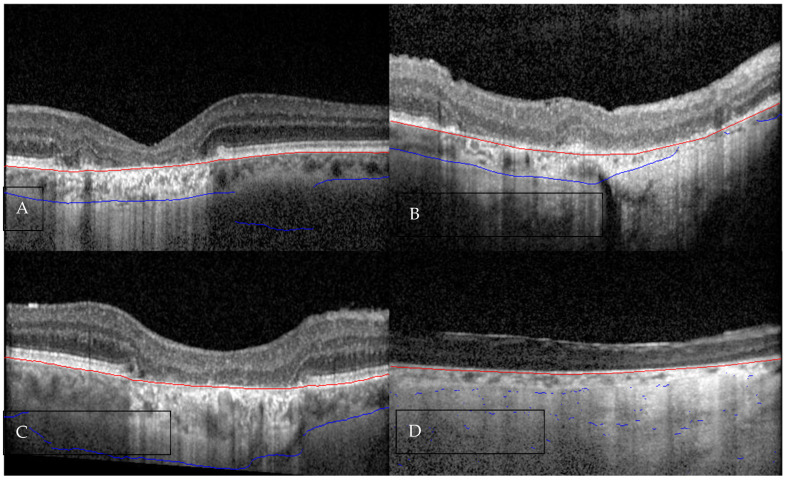
Representative OCT B-scans illustrating varying degrees of inaccuracy in automated segmentation of the choroidal outer boundary (COB) (blue line). The inner choroidal boundary (red line) appears to be accurately segmented in all the images (**A**) Minor inaccuracy (<33%), beginning at the junction of the hyper-transmission area. (**B**) Moderate inaccuracy (33–66%), observed in regions of marked choroidal thinning starting from the entry of a posterior ciliary vessel. (**C**) Major inaccuracy (>66%), starting at the junction of the hyper-transmission area. (**D**) Near-complete segmentation failure (approximately 100% inaccuracy), associated with severe choroidal thinning and poor image quality.

Performance was assessed by comparing automated segmentations against manual ground truth with slice-wise error analysis. Each B-scan’s boundary deviation was graded: 0 = accurate, 1 = minor (≤33% of the B-scan deviated), 2 = moderate (33–66%), 3 = major (>66% or missed) (Figure 2). The percentage quantified the portion of each B-scan length over which the automated boundary showed a visually discernible displacement from the manual ground truth. We used this four-level ordinal scheme to provide a clinically interpretable summary of error extent and to remain robust in GA scans where pixel-distance metrics can be unstable when the boundary cue is intermittently ambiguous. To balance efficiency with mandatory human review time, this grading served as a standardized correction trigger: Grade 2–3 deviations required manual correction, whereas Grade 1 deviations underwent rapid visual verification and were corrected only when involving the COB near hyper-transmission transitions or in low-signal or motion-affected scans. This grading represents an expert, clinically oriented qualitative assessment rather than a pixel-level quantitative agreement metric; formal statistical comparisons (e.g., Dice similarity or boundary distance) were not the focus of this failure-mode analysis. This COB-focused trigger reflects that hyper-transmission in GA preferentially reduces contrast at the CSI, whereas the CIB remains anchored to the higher-contrast RPE–Bruch’s membrane complex (Table 1). Manual segmentation paid close attention to distinguishing the true scleral interface from hyper-transmission artifacts. Manual ground-truth delineation followed a consistent rule of selecting the most anatomically plausible CSI/COB based on continuity across adjacent B-scans and avoiding boundary drift into hyper-transmission “tailing” or washout regions. We also identified error causes such as low image quality, motion, or lesion morphology.

We computed confusion matrices and derived accuracy, sensitivity, precision, and F_1_ score for CIB and COB. Weighted F1 was computed using support-weighting, where each label’s F_1_ is weighted by its number of ground-truth instances. Specificity and negative predictive value were not reported since every B-scan contains true CIB/COB boundaries. Metrics were calculated separately for CIB and COB to assess performance under different error tolerance levels. For tolerance mapping, we reported (i) a strict criterion where only Grade 0 was treated as acceptable and (ii) a lenient criterion where Grades 0–1 were treated as acceptable; Grades ≥ 2 were treated as failures in both settings. Inter-reader agreement for the assigned inaccuracy grades was quantified using ICC (two-way mixed-effects model, absolute agreement).

### 2.5. Workflow Efficiency Assessment

To evaluate the clinical utility of the AI model despite its limitations, we compared the time required for two workflows in three randomly selected volumes (3 × 97 B-scans): “Manual-Only” segmentation—the time taken for an expert to segment the full choroidal volume (CIB and COB) from scratch; “AI-Assisted” workflow—the time taken for the model to generate initial boundaries for each B-scan (CIB and COB) plus the time required for a human expert to verify and correct all segmentation errors (specifically correcting COB displacements in areas of signal washout). COB-specific corrections were standardized as segment-level edits restricted to regions where the COB visibly drifted into hyper-transmission “tailing” or where CSI visibility was locally ambiguous. Corrections were considered complete when the overlaid boundaries were anatomically plausible and continuous across adjacent B-scans throughout the volume. Timing was recorded by an experienced grader using a stopwatch, from the start of each workflow to completion, and summarized across volumes.

### 2.6. Statistical Analysis

Normality was tested with the Shapiro–Wilk test. The ANOVA test was used for normally distributed data and the Kruskal–Wallis test for non-normally distributed data. Accordingly, timing results were summarized as mean ± SD when normally distributed and as median (IQR) when non-normally distributed. The chi-square test was employed to compare the two methods. The chi-square test was employed to compare categorical error outcomes, specifically the distribution of slice-wise deviation grades (0–3) and the proportion of acceptable vs unacceptable segmentations under predefined tolerance thresholds (e.g., Grade 0–1 vs. ≥2), which reflect the expected manual correction burden. A confidence interval (CI) of 95% and a *p*-value of ≤0.05 were considered statistically significant. All statistical procedures were executed using IBM SPSS Statistics software, version 26.

## 3. Results

### 3.1. Choroidal Boundary Segmentation Performance and Failure Analysis

Across 5723 B-scans, CIB was highly robust, with a strict accuracy (Grade 0) of 94.8%, a weighted F_1_ score of 0.97, and a precision of 1.0. The remaining were minor deviations at 2.5%, and moderate and major errors at 1.01% and 1.64%, respectively.

**Figure 3:**
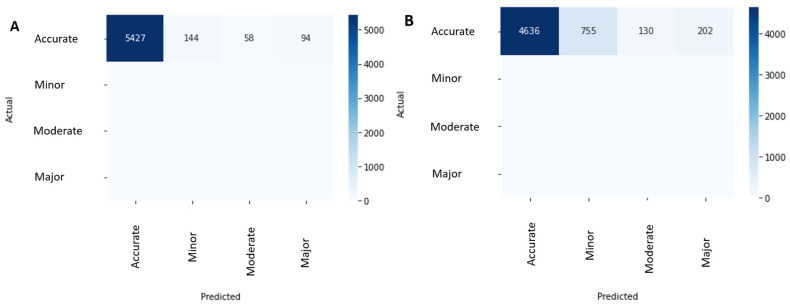
Confusion matrices for choroidal boundary grading. (**A**) Choroidal inner boundary (CIB) and (**B**) choroidal outer boundary (COB) using a four-level error scale: Accurate, Minor (≤33% of the B-scan deviated), Moderate (33–66%), Major (>66% or missed). Rows denote ground-truth class and columns denote model predictions. Because a true anatomical boundary exists on every slice, all reference labels fall in the “Accurate” row. Counts shown in each cell indicate the number of B-scans assigned to each predicted category (CIB: 5427 Accurate, 144 Minor, 58 Moderate, 94 Major; COB: 4636 Accurate, 755 Minor, 130 Moderate, 202 Major), illustrating that most slices were classified as error-free and that most residual errors were minor.

In contrast, COB accuracy dropped to 81.0% (weighted F_1_ score: 0.89). In this strict setting, the lower COB weighted F_1_ score was primarily driven by reduced recall, meaning more missed accurate localizations, whereas precision remained high, indicating few false positives. There were minor deviations in 13.2% of scans and moderate-to-major errors in about 6% (Categories 2 and 3). Notably, several COB errors appeared smoothly after probability-to-boundary conversion and smoothing but were clinically meaningful on overlay inspection and therefore required manual correction. Confusion matrices (Figure 3) indicate many more Grade-3 errors for COB (202 scans) compared to CIB (94 scans). However, considering minor deviations (≤33% deviation is considered acceptable) increased COB sensitivity to 94.2%, suggesting many errors consisted of minor spatial deviation rather than outright failures. Inter-grader reliability for identifying the number of accurate B-scans per volume was excellent, with ICCs of 0.938 (95% CI: 0.874–0.970, *p* < 0.001) for COB and 0.923 (95% CI: 0.847–0.962, *p* < 0.001) for CIB.

### 3.2. Failure Mode Characterization

The causes of inaccuracy are detailed in Table 1. While CIB errors were primarily driven by image quality issues, COB failures were largely attributed to hyper-transmission artifacts specific to GA area. Specifically, signal washout and hyper-transmission defects accounted for approximately 40% of COB errors, where the increased light penetration through the atrophic RPE obscured CSI. Moreover, CIB errors were most frequently associated with superficial signal loss or low image quality and motion, whereas COB errors most frequently involved hyper-transmission and signal washout and vessel-related confounders (Table 1) [12]. These “acceptable” (≤33%) COB deviations typically reflected subtle boundary drift in segments where the CSI is intrinsically low-contrast and is locally biased by vessel-related streaks or spatially variable deep signal (hyper-transmission adjacent to attenuation), yielding a smooth but slightly offset outer boundary.

### 3.3. Workflow Efficiency and Clinical Utility

Despite the limitations in COB accuracy requiring human verification, the AI-assisted workflow demonstrated a dramatic improvement in efficiency compared to manual grading. Manual-only segmentation of the full choroid (CIB and COB) across a complete 97-slice volume was extremely labor-intensive, requiring an average of approximately 7 h per volume by a single experienced ophthalmologist. In contrast, the “AI-assisted” workflow, comprising automated inference followed by expert correction of artifacts, took an average of 45 min per volume by a single experienced ophthalmologist. Although the model frequently misidentified the COB in areas of hyper-transmission (necessitating human correction), the AI provided a foundational structure that reduced the total human effort by approximately 90%.

## 4. Discussion

In this study, we performed a failure analysis of automated choroidal boundary detection in GA eyes using a validated choroid segmentation tool (NMI ChoroidAI), focusing on the markedly different behavior of the model when estimating the CIB versus the COB. While the CIB remained highly detectable even in advanced GA (94.8% Grade-0 accuracy), COB definition consistently deteriorated in areas of hyper-transmission, reaching only 81.0% strict accuracy.

Compared with prior studies, which have predominantly evaluated choroidal boundary detection in healthy eyes or non-GA AMD, our findings demonstrate a critical gap in the literature that is current automated choroidal segmentation methods rarely account for GA-specific optical artifacts. Several high-performing deep-learning models have reported strong choroidal and retinal boundary accuracy under normal imaging conditions [10,11], yet these results do not necessarily translate to GA eyes, where loss of the RPE results in marked hyper-transmission and signal washout. To further contextualize this gap, Table 2 summarizes recent automated segmentation literature. As highlighted, while recent deep learning models achieve excellent performance, they have predominantly been validated on healthy cohorts or focused on shallower retinal layers. In contrast, our study uniquely targets the COB in GA, directly addressing the disease-specific optical challenges that limit autonomous segmentation. Consistent with this, recent research on GA imaging has emphasized that RPE loss fundamentally alters OCT contrast characteristics [12] which result in producing structural ambiguities at the CSI that complicate automated COB estimation. Our strict COB accuracy of 81% reflects a pathology-driven limitation rather than a model flaw alone. However, when scans with minor inaccuracies were included, the model demonstrated improved performance, achieving an overall accuracy of 94.2%. Only 13.2% of scans required minor manual corrections, substantially reducing the time required and overall workload.

Accurate and reproducible delineation of the choroidal inner and outer boundaries is clinically crucial; as choroidal thickness and vascular metrics are increasingly investigated as imaging biomarkers and potential exploratory endpoints in AMD and GA research, as well as interventional trials [2,17,18]. Minor COB deviations primarily alter the estimated outer contour and therefore can bias choroidal thickness and area-based biomarkers. For ratio-based measures such as CVI, small smooth shifts may be partly buffered, but they can still introduce longitudinal bias if the shift is not consistent across visits [2,4,17,18]. Accordingly, quantitative choroidal biomarkers should be derived only after manual verification, and, when needed, correction, of the segmented boundaries. We do not recommend proceeding with CT/CVI measurements from fully automated segmentations in GA eyes without expert review. Manual slice-by-slice segmentation is prohibitive in volume, explaining why AI-assisted workflows are appealing. In this study, AI-assisted processing reduced the total time to about 45 min per volume, representing nearly a 90% reduction in human effort. This pattern is consistent with prior AI-based OCT pipelines, which have demonstrated substantial time savings in clinical trial settings while still relying on human verification rather than fully autonomous deployment [19,20,21]. In this context, our model should therefore be considered as a workflow accelerator, providing a plausible anatomical estimate whose COB accuracy requires expert verification before use in trials.

The strong performance on the CIB and the weaker performance on the COB arise from fundamentally different signal characteristics within SD-OCT volumes. The CIB corresponds to a high-contrast RPE–Bruch’s membrane transition, which remains anatomically recognizable even in eyes with advanced cRORA [12,22]. This structural stability allows deep networks to detect the CIB reliably across varying image quality and patient-dependent fluctuations [10]. This reflects the fact that the CIB is anchored to a sharp inner boundary cue at the RPE–Bruch’s membrane complex that often remains identifiable even in advanced atrophy. In contrast, COB estimation depends on consistent visibility of the choroidoscleral interface, which is more vulnerable to deep-signal variability in SD-OCT than in swept-source OCT volumes with improved depiction of deeper structures [15]. Consistent with this, hyper-transmission accounted for ~40% of COB errors, whereas CIB inaccuracies were predominantly driven by more superficial signal loss and image-quality limitations (Table 1). Because the COB often represents a gradual low-contrast transition rather than a discrete interface in GA eyes, the model encounters regions where no clear boundary exists, making errors unavoidable regardless of architectural complexity. Compared with healthy retinas, GA-related RPE loss produces hyper-transmission and variable deep-signal attenuation that destabilize CSI visibility and degrades COB delineation. Accordingly, we anticipate that SS-OCT, with improved depiction of deeper structures, may further enhance COB/CSI delineation, particularly in eyes with thicker choroids where SD-OCT penetration can be more limited. Notably, GA eyes in our cohort typically exhibited relatively thin choroids, and the CSI and posterior choroidal boundary were generally visible on SD-OCT, supporting the feasibility of SD-OCT-based analysis in this setting.

Another factor influencing performance is the directionality of OCT signal propagation. The hyper-transmission that accompanies RPE loss increases light penetration into deeper tissues but simultaneously reduces the local contrast between the choroid and sclera. This “signal dilution” effect decreases the sharpness of the true boundary and increases the probability that the model converges on an anatomically invalid but visually dominant tailing artifact. This behavior is consistent with histopathologic and imaging observations describing the fading of the CSI in cRORA [12]. These effects can also influence boundary consistency across adjacent B-scans: while slice-to-slice stabilization steps may improve the continuity of the CIB, they offer limited benefit for the COB because its visibility fluctuates along the scan. Some preprocessing strategies have been reported to stabilize retinal and choroidal boundaries in automated OCT analysis [10,11]. In regions where hyper-transmission abruptly increases or posterior ciliary vessels locally dominate the intensity profile, preprocessing cannot reconstruct a boundary that is physically absent.

NMI ChoroidAI tool integrates manual editing within the automated segmentation workflow, allowing users to refine results in real time while viewing OCT images. Importantly, corrections are required only for boundary segments that deviate from the true anatomical layer, rather than redrawing the entire contour. This selective adjustment approach greatly improves efficiency and enables rapid generation of high-quality segmentations.

Furthermore, architectural elements such as residual connections and attention mechanisms help the model focus on coherent, layer-specific patterns rather than isolated noise. Residual learning has been shown to improve detection of fine structural transitions in medical image segmentation [23,24], while attention mechanisms enhance the network’s ability to prioritize relevant anatomical features and suppress background clutter [16,25,26]. While this improves CIB detection, it also means the model tends to ignore faint or discontinuous deep signals, which in GA lesions are often the only remaining cues for COB localization. Thus, architecture inadvertently reinforces the performance gap: robust for structures with preserved reflectivity, fragile for structures whose visibility deteriorates under disease-specific optical conditions.

### 4.1. Limitations

Several limitations of this study should be acknowledged. First, the analysis was performed exclusively on eyes with established GA that met classification of atrophy meetings (CAM) criteria for cRORA. Earlier stages such as iRORA, which exhibit only partial RPE-photoreceptor disruption, were not included. Because the optical visibility of the CSI degrades progressively along the iRORA to cRORA continuum, the present model may not generalize to earlier atrophic stages where the boundary is partially preserved and the pattern of hyper-transmission differs [22].

Second, although the model demonstrated high reliability for the CIB, COB performance was strongly constrained by OCT physics rather than by network architecture. In regions of pronounced hyper-transmission, the choroid–sclera interface becomes attenuated or disappears entirely, resulting in true signal ambiguity. As described in structural and histopathologic studies of GA, this washout of the deeper interface is an inherent consequence of RPE loss [12]. In such regions, even a theoretically ideal model would lack sufficient reflectivity cues to localize the COB consistently, limiting the achievable performance ceiling.

Third, the dataset consisted of SD-OCT volumes from a single imaging platform (Heidelberg Spectralis), all with relatively high acquisition quality. Device-specific differences such as axial resolution, sensitivity roll-off, and noise characteristics may affect the appearance of the COB. Previous work has shown that automated OCT algorithms may degrade when transferred across devices without retraining [9]. Broader validation across additional devices, including swept-source OCT with deeper penetration profiles, will therefore be required.

Finally, all segmentations were evaluated in a curated dataset without the full range of real-world image artifacts (motion, low SNR, shadowing from media opacities). While registration and curvature normalization can reduce inter-scan variability, they cannot reconstruct a boundary that is not visible due to washout. Consequently, automated COB estimation in lower-quality OCT scans may be more error-prone. In eyes with more severe signal washout or extensive hyper-transmission, COB ambiguity is more frequent and corrections may take longer; therefore, the reported time-efficiency may not fully generalize to lower-quality or more advanced cases.

### 4.2. Prospective Trajectories

Future work should focus on addressing the optical limitations that underline COB failures. Because the CSI progressively fades along the CAM-defined iRORA to cRORA spectrum, incorporating stage-specific annotations may help models learn how boundary visibility deteriorates with advancing atrophy in GA patients [22]. Additional progress will require artifact-aware or physics-informed approaches capable of distinguishing true boundary loss from hyper-transmission-related washout [12]. Broader training across multiple OCT platforms is also needed, as automated algorithms often show reduced performance when transferred between devices [9]. In addition, multimodal integration such as SS-OCT for improved deep choroidal penetration and OCT angiography for complementary vascular context may further improve COB detectability and should be evaluated in dedicated multimodal studies. Together, these steps may reduce analysis time further, but human verification will still be required for clinical-grade outputs.

## 5. Conclusions

This study provides a failure analysis of automated choroidal boundary detection in GA eyes imaged with Spectralis SD-OCT. While the model reliably identified the CIB, COB detection was substantially limited by GA-related optical artifacts, particularly hyper-transmission in cRORA lesions. Despite these inaccuracies, the AI-assisted workflow reduced segmentation time from approximately seven hours to forty-five minutes per volume (1/10th), underscoring its value as an efficiency tool rather than an autonomous solution. Improved handling of deep signal loss, stage-specific modeling across the iRORA–cRORA spectrum, and broader cross-device training will be required before automated COB measurements can be used reliably in future GA studies.

## Figures and Tables

**Table 1:** Causes of inaccuracy in each boundary.

Choroidal inner boundary		
Low image quality/signal loss	~45%	Degraded scan contrast, media opacities, or defocus led to blurred definition of the RPE–choroid interface.
Sudden change in RPE curvature	~30%	Abrupt topographic variations in the RPE, particularly in parafoveal regions or near drusen, caused local mis-registration of the CIB line.
Fixation change/motion artifact	~20%	Horizontal eye movement between adjacent B-scans slightly displaced the inner choroidal contour, particularly near the disk margin.
Combined effects (e.g., curvature + signal drop-off)	~5%	Compounded quality issues or irregular RPE slopes caused limited regional deviations.
Choroidal outer boundary		
Signal washout/Hyper-transmission	~40%	Hyper-transmission artifacts beneath the atrophic RPE caused the choroidal–scleral interface to fade or become indistinguishable from the sclera.
Fixation change/motion artifact	~25%	Minor eye movement between scans distorted local curvature of the choroid–sclera interface.
Presence of large posterior ciliary vessels	~20%	These vessels create local discontinuities or high-contrast streaks that confuse the model’s estimate of the scleral border.
Combined low quality + motion	~15%	Concurrent factors caused larger regional deviations (>66%).

**Table 2:** Comparison of the proposed AI-assisted workflow with recent OCT segmentation studies.

Study (Year)	Target Population	Segmentation Target	Imaging Modality	Methodology	Key Findings and Relevance to Our Study
Current study (2026)	GA	COB, CIB	SD-OCT	ResUNet + Mandatory Human Verification	CIB Accuracy: 94.8%; Strict COB Accuracy: 81.0% (Lenient: 94.2%). Demonstrates that GA-specific hyper-transmission inherently limits autonomous COB detection
Valsecchi et al. (2025) [7]	Healthy Eyes	Choroidal Vessels	SD-OCT	Semi-automated Deep Learning	Achieved robust vascular mapping in normal eyes; however, lacks validation under GA conditions where deep-signal washout obscures boundaries.
Arora et al. (2024) [6]	Healthy Eyes	Choroidal Contour	SD-OCT	Automated 3D Mapping	Provided precise 3D choroidal contours in healthy cohorts does not account for GA-specific RPE loss and signal attenuation.
Cao et al. (2024) [16]	Various Pathologies	Retinal Layers	SD-OCT	Self-attention CNN	High accuracy in superficial retinal layers but did not address the deeper choroidoscleral interface (COB) which degrades in advanced atrophy.
Vupparaboina et al. (2023) [15]	General/Healthy	Choroidal Vasculature	SS-OCT	Phansalkar Thresholding	Utilized SS-OCT for deeper tissue penetration, highlighting the physical limits of SD-OCT (used in our study) for deep choroidal features in pathology.

GA, geographic atrophy; CIB, choroidal inner boundary; COB, choroidal outer boundary; SD-OCT, spectral-domain optical coherence tomography; SS-OCT, swept-source optical coherence tomography; CNN, convolutional neural network; 3D, three-dimensional.

## Data Availability

Data availability may be provided upon reasonable request to the corresponding author.

## Abbreviations

The following abbreviations are used in this manuscript:

GA	Geographic atrophy
SD-OCT	Spectral domain optical coherence tomography
CIB	Choroidal inner boundary
COB	Choroidal outer boundary
NMI	NetraMind Innovations
AI	Artificial Intelligence
AMD	Age related macular degeneration
RPE	Retinal pigment epithelium
CSI	Choroidoscleral interface
ICC	Intraclass coefficient
cRORA	complete RPE and outer retinal atrophy
CAM	Classification of Atrophy Meetings

## References

[1] Wong W.L., Su X., Li X., Cheung C.M.G., Klein R., Cheng C.Y., Wong T.Y. (2014). Global prevalence of age-related macular degeneration and disease burden projection for 2020 and 2040: A systematic review and meta-analysis. Lancet Glob. Health.

[2] Sadeghi E., Valsecchi N., Rahmanipour E., Ejlalidiz M., Hasan N., Vupparaboina K.K., Ibrahim M.N., Rasheed M.A., Baek J., Iannetta D. (2025). Choroidal biomarkers in age-related macular degeneration. Surv. Ophthalmol..

[3] Takahashi A., Ooto S., Yamashiro K., Tamura H., Oishi A., Miyata M., Hata M., Yoshikawa M., Yoshimura N., Tsujikawa A. (2018). Pachychoroid Geographic Atrophy: Clinical and Genetic Characteristics. Ophthalmol. Retina.

[4] Sacconi R.M., Battista M., Borrelli E.M., Senni C., Tombolini B., Grosso D., Querques L., Bandello F.M., Querques G. (2022). Choroidal vascularity index is associated with geographic atrophy progression. Retina.

[5] Querques G., Costanzo E., Miere A., Capuano V., Souied E.H. (2016). Choroidal Caverns: A Novel Optical Coherence Tomography Finding in Geographic Atrophy. Investig. Ophthalmol. Vis. Sci..

[6] Arora S., Singh S.R., Rosario B., Ibrahim M.N., Selvam A., Zarnegar A., Harihar S., Sant V., Sahel J.A., Vupparaboina K.K. (2024). Three-dimensional choroidal contour mapping in healthy population. Sci. Rep..

[7] Valsecchi N., Sadeghi E., Davis E., Ibrahim M.N., Hasan N., Bollepalli S.C., Singh S.R., Fontana L., Sahel J.A., Vupparaboina K.K. (2025). Assessment of choroidal vessels in healthy eyes using 3-dimensional vascular maps and a semi-automated deep learning approach. Sci. Rep..

[8] Al-Khersan H., Sodhi S.K., Cao J.A., Saju S.M., Pattathil N., Zhou A.W., Choudhry N., Russakoff D.B., Oakley J.D., Boyer D. (2025). Deep Learning-Based Segmentation of Geographic Atrophy: A Multi-Center, Multi-Device Validation in a Real-World Clinical Cohort. Diagnostics.

[9] Zhang G., Fu D.J., Liefers B., Faes L., Glinton S., Wagner S., Struyven R., Pontikos N., A Keane P., Balaskas K. (2021). Clinically relevant deep learning for detection and quantification of geographic atrophy from optical coherence tomography: A model development and external validation study. Lancet Digit. Health.

[10] Mazzaferri J., Beaton L., Hounye G., Sayah D.N., Costantino S. (2017). Open-source algorithm for automatic choroid segmentation of OCT volume reconstructions. Sci. Rep..

[11] Niu S., de Sisternes L., Chen Q., Leng T., Rubin D.L. (2016). Automated geographic atrophy segmentation for SD-OCT images using region-based CV model via local similarity factor. Biomed. Opt. Express.

[12] Fleckenstein M., Mitchell P., Freund K.B., Sadda S., Holz F.G., Brittain C., Henry E.C., Ferrara D. (2018). The Progression of Geographic Atrophy Secondary to Age-Related Macular Degeneration. Ophthalmology.

[13] Derradji Y., Mosinska A., Apostolopoulos S., Ciller C., De Zanet S., Mantel I. (2021). Fully-automated atrophy segmentation in dry age-related macular degeneration in optical coherence tomography. Sci. Rep..

[14] Klein R., Meuer S.M., Knudtson M.D., Klein B.E. (2008). The epidemiology of progression of pure geographic atrophy: The Beaver Dam Eye Study. Am. J. Ophthalmol..

[15] Ibrahim M.N., Bollepalli S.C., Selvam A., Sant V., Harihar S., Sahel J.A., Chhablani J., Vupparaboina K.K. Accurate Detection of 3D Choroidal Vasculature Using Swept-Source OCT Volumetric Scans Based on Phansalkar Thresholding. Proceedings of the 2023 IEEE EMBS International Conference on Biomedical and Health Informatics (BHI).

[16] Cao G., Wu Y., Peng Z., Zhou Z., Dai C. (2024). Self-attention CNN for retinal layer segmentation in OCT. Biomed. Opt. Express.

[17] Sadda S.R., Chakravarthy U., Birch D.G., Staurenghi G., Henry E.C., Brittain C. (2016). Clinical endpoints for the study of geographic atrophy secondary to age-related macular degeneration. Retina.

[18] Vallino V., Berni A., Coletto A., Serafino S., Bandello F., Reibaldi M., Borrelli E. (2024). Structural OCT and OCT angiography biomarkers associated with the development and progression of geographic atrophy in AMD. Graefe’s Arch. Clin. Exp. Ophthalmol..

[19] Enzendorfer M.L., Tratnig-Frankl M., Eidenberger A., Schrittwieser J., Kuchernig L., Schmidt-Erfurth U. (2025). Rethinking Clinical Trials in Age-Related Macular Degeneration: How AI-Based OCT Analysis Can Support Successful Outcomes. Pharmaceuticals.

[20] Fu D.J., Glinton S., Lipkova V., Faes L., Liefers B., Zhang G., Pontikos N., McKeown A., Scheibler L., Patel P.J. (2024). Deep-learning automated quantification of longitudinal OCT scans demonstrates reduced RPE loss rate, preservation of intact macular area and predictive value of isolated photoreceptor degeneration in geographic atrophy patients receiving C3 inhibition treatment. Br. J. Ophthalmol..

[21] Rahmanipour E., Afazel S., Ashrafi S., Sadeghi E., Ghorbani M., Zarranz-Ventura J., Chhablani J. (2025). Artificial intelligence in ophthalmology clinical trials: A narrative review. Graefe’s Arch. Clin. Exp. Ophthalmol..

[22] Guymer R.H., Rosenfeld P.J., Curcio C.A., Holz F.G., Staurenghi G., Freund K.B., Schmitz-Valckenberg S., Sparrow J., Spaide R.F., Tufail A. (2020). Incomplete Retinal Pigment Epithelial and Outer Retinal Atrophy in Age-Related Macular Degeneration: Classification of Atrophy Meeting Report 4. Ophthalmology.

[23] He K., Zhang X., Ren S., Sun J. Deep residual learning for image recognition. Proceedings of the IEEE Conference on Computer Vision and Pattern Recognition.

[24] Sahoo J., Saini S.K., Singh S., Saxena A.K., Sharma S., Awasthi A., Rajalakshmi R. (2024). Residual learning for segmentation of the medical images in healthcare. Meas. Sens..

[25] Soni T., Gupta S., Almogren A., Altameem A., Rehman A.U., Hussen S., Bharany S. (2025). ARCUNet: Enhancing skin lesion segmentation with residual convolutions and attention mechanisms for improved accuracy and robustness. Sci. Rep..

[26] Xie Y., Yang B., Guan Q., Zhang J., Wu Q., Xia Y. (2023). Attention mechanisms in medical image segmentation: A survey. arXiv.

